# Low-Dose Creatine Supplementation May Be Effective in Early-Stage Statin Myopathy: A Preliminary Study

**DOI:** 10.3390/jcm13237194

**Published:** 2024-11-27

**Authors:** Elena Scarsi, Ulrico Dorighi, Enrico Adriano, Marina Grandis, Maurizio Balestrino

**Affiliations:** 1 Dipartimento di Neuroscienze, Riabilitazione, Oftalmologia, Genetica e Scienze Materno-Infantili (DINOGMI), University of Genoa, 16126 Genoa, Italy; elena.scarsi1994@gmail.com (E.S.); adriano@neurologia.unige.it (E.A.); marina.grandis@unige.it (M.G.); 2 IRCCS Ospedale Policlinico San Martino, 16132 Genoa, Italy; ulrico.dorighi@hsanmartino.it

**Keywords:** creatine, statin, myopathy, therapy

## Abstract

**Background.** Statins are the main cholesterol-lowering treatments, but often they are stopped because of statin myopathy. Expensive second-line treatments are then prescribed, causing a burden on the health system. Previous research showed that creatine supplementation may be a relatively inexpensive, safe, and effective way to mitigate statin toxicity to the muscle. **Methods.** We thus investigated the tolerability and effectiveness of creatine supplementation in consecutive patients with statin myopathy, as observed at our Cardiology or Neurology outpatient services for previous cardiac or cerebral ischemic disease. **Results.** We confirmed previous findings showing that creatine supplementation is safe and well tolerated even in this elderly population. Eleven of the thirteen enrolled patients completed the study, and only one patient interrupted the study because of a creatine-related issue (elevation of serum creatinine). Creatine supplementation significantly reduced the Shewmon and Craig’s “myopathy score”, while it did not reduce serum creatine kinase (CK), a marker of muscle structural damage. Notably, creatine supplementation was effective at a dose of 1 g. t.i.d., lower than usually prescribed in the international literature and within the recommendations of health agencies like the Italian Ministry of Health. **Conclusion.** Creatine supplementation may improve statin myopathy in its milder and/or earlier form when serum CK is not elevated. Since creatine is relatively inexpensive, its supplementation may be used instead of switching from statins to the very expensive second-line anti-cholesterol treatments.

## 1. Introduction

Hypercholesterolemia is a major risk factor in atherosclerosis, and guidelines recommend its aggressive reduction to prevent severe diseases like myocardial infarction, ischemic stroke, and more [1]. Statins (HMG-CoA reductase inhibitors) are a cornerstone of this reduction, their use being highly recommended [2]. However, statin-associated myopathy is a frequent side effect, whose frequency has been estimated in 10–25% of the cases and which often forces patients to stop taking statin medication [3,4]. Several changes in treatment may be operated to reduce this toxicity, e.g., decreasing the dose of the statin, taking statins on alternate days, or trying a different statin. Some of these, like decreasing the dose or the frequency of statin assumption, carry the risk of not meeting the LDL-cholesterol target concentration. Moreover, even after these changes in treatment, many subjects need to stop statin assumption and move to a second-line anti-cholesterol treatment. Currently, several alternative treatments are available in cases of statin intolerance, including ezetimibe, anti-PCSK9 monoclonal antibodies, inclisiran, and bempedoic acid [5,6]. However, non-statin treatments are expensive, and their widespread use would probably not be sustainable for health systems, both those of developing countries and of high-income countries as well [7,8,9]. As an example, Table 1 shows the cost of some cholesterol-lowering treatments in Italy. Our group and others have shown that creatine, a widespread nutritional supplement, is able to counter statin toxicity and prevent or reduce statin-associated myopathy [10,11,12,13,14,15]. The supplementation of creatine is safe [11]; moreover, one month of creatine supplementation at the dose of 3 g/day (the dose we used in the present study) costs in Italy about €15/month (see, for example, [16]). Thus, creatine supplementation may represent an alternative to expensive cholesterol-lowering drugs in some cases of statin-associated myopathy. From this point of view, an issue to be considered is that creatine is usually administered at relatively high doses; for example, Shewmon and Craig, in the paper that initially reported its effectiveness in statin myopathy, used creatine 5 g twice daily for 5 days (creatine loading) followed by creatine 5 g/day as a continuation. In Italy, the Ministry of Health recommends creatine supplementation to the maximum dose of 6 g/day for one month and 3 g/day as a long-term therapy [17]. Thus, we carried out this preliminary study to investigate if such a relatively low dose of creatine might be sufficient to counter the symptoms of statin-associated myopathy.

## 2. Materials and Methods

The study was preliminarily approved by our regional Ethics Committee.

(A) Selection of study subjects

Patients were referred from the outpatient services of either the Cardiology or the Neurology outpatient services of our hospital. Patients had to be intolerant to at least one statin to enter the trial (see Table 2). All patients were under the care of the outpatient service for the prevention of cardiovascular or cerebrovascular ischemic disease or both. Table 2 lists the inclusion and exclusion criteria and the preset criteria for study withdrawal.

(B) Study design

Serum creatinine, cholesterol, LDL-cholesterol, and creatine kinase (CK) were assessed at baseline, as was creatinine clearance (Cockcroft-Gault formula). After enrollment, the statin was stopped, and creatine was administered at a loading dose of 2 g t.i.d. for 7 days. Afterwards, the same statin was resumed at the same dosage, and creatine supplementation was continued at the dose of 1 g t.i.d. for 4 months. Patients were visited each month. Other than for specific study procedures, all patients were cured according to routine protocols and treatments. At each visit, history was taken, a physical and a neurological examination were carried out, and a survey of muscle symptoms was performed. Additionally, at each visit, the Shewmon and Craig’s “myopathy score” [10] was calculated. The latter takes into consideration muscle pain, muscle weakness, and cramp severity (i.e., cramps frequency, duration, and painfulness). The severity of each symptom is gauged by the patient in a 0–10 range by drawing a cross along a horizontal line, graduated from 0 to 10. All scores had to be whole numbers; decimals were not allowed. The sum of the three scores was the “myopathy score”. Additionally, we carried out the following biochemical evaluations: at the month 1 visit, serum creatinine; at month 2, serum creatinine, total cholesterol, and LDL-cholesterol, CK; at month 3, serum creatinine; at month 4, serum creatinine, total cholesterol, LDL-cholesterol and CK. All analyses were carried out by the clinical laboratory of our hospital using routine equipment and reagents. The primary endpoint was to verify whether at least half of the patients treated with creatine and the statin, to which they were intolerant, completed the study. Secondary endpoints were as follows: (1) the difference in Shewmon and Craig’s “myopathy scores” between the baseline and visit 4, and (2) the difference in serum CK values between the baseline and visit 4. All patients were given a commercially available preparation (Novacrea^®^ manufactured by Novaneuro srl, Genoa, Italy), containing 1 g creatine monohydrate, 0.5 g honey, and excipients in the form of chewable tablets.

(C) Statistical analysis

Statistical analysis was performed using GraphPad Prism version 10.0.0 for Windows, GraphPad Software, Boston, MA, USA, www.graphpad.com accessed on 1 September 2024. We chose to use parametric statistics because the small size of the sample does not allow us to assume a gaussian distribution. A probability value (“*p* value”) of 0.05 was used to indicate significance.

## 3. Results

### 3.1. Patients, General Data

A total of twenty-one patients were screened, thirteen females and eight males, aged 53 to 81 years. Of these,

⁻ five patients were excluded at screening because they did not meet inclusion criteria;
⁻ two patients were excluded at screening because they were assuming red rice supplements instead of statins.

Of the remaining fourteen patients that were enrolled, three patients had to be withdrawn from the study for the following reasons: one male patient was withdrawn after the screening evaluation, before the beginning of the study treatment, because serum CK rose to >1200 U/L; one female patient was withdrawn because of gastrointestinal symptoms that occurred after two months supplementation; and such symptoms were of rather acute onset and were judged to be of infectious (probably viral) etiology, unrelated to creatine supplementation. Another female patient was withdrawn due to the worsening of renal function above 1.5 times the ULN of creatinine (see preset criteria for study withdrawal in Table 2).

**Figure 1:**
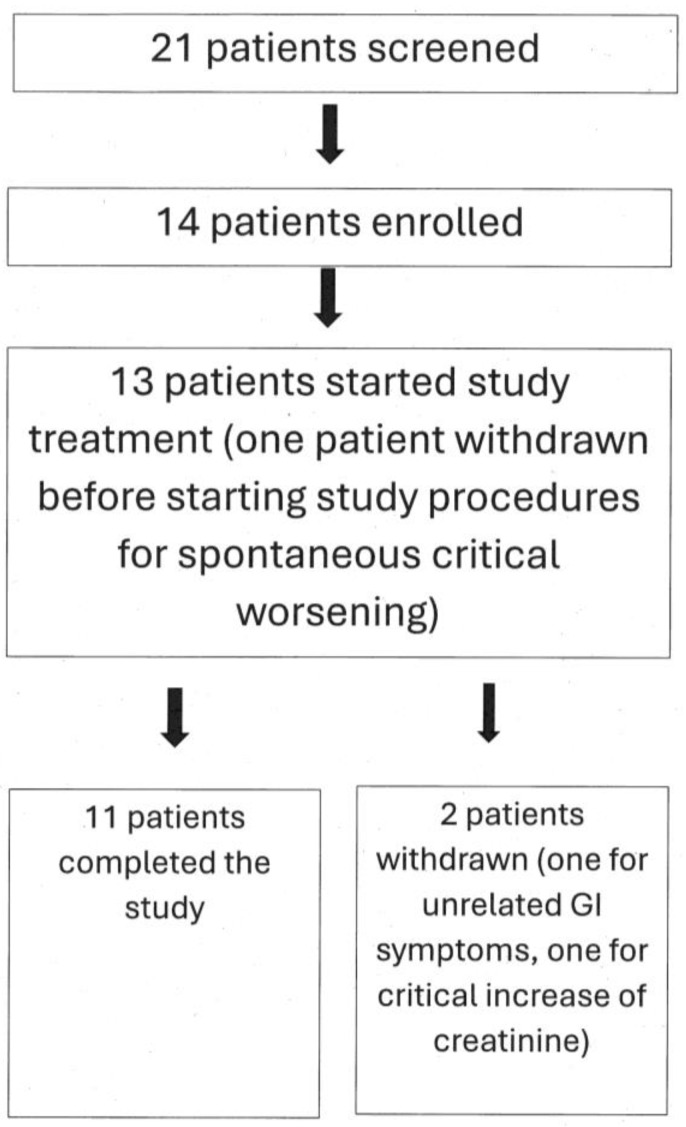
Flow chart depicting study patients’ numbers. See text for additional explanations.

Finally, 11 enrolled patients completed the study. Of them, six were taking Rosuvastatin, four were taking Atorvastatin, and one was taking Simvastatin. Figure 1 graphically represents these data.

Four patients were taking statin in the primary prevention of cardiovascular or cerebrovascular diseases; five patients were taking statin in secondary prevention for ischemic heart disease (all had myocardial infarction and coronary stent placement); and two patients were taking statin for secondary prevention of ischemic stroke (one had middle cerebral artery occlusion and one had Percheron’s artery occlusion).

The most frequent comorbidities were high blood pressure, overweight, and diabetes mellitus. None had a personal nor family history of neuromuscular diseases.

### 3.2. Creatinine and Cholesterol Dosages

**Figure 2:**
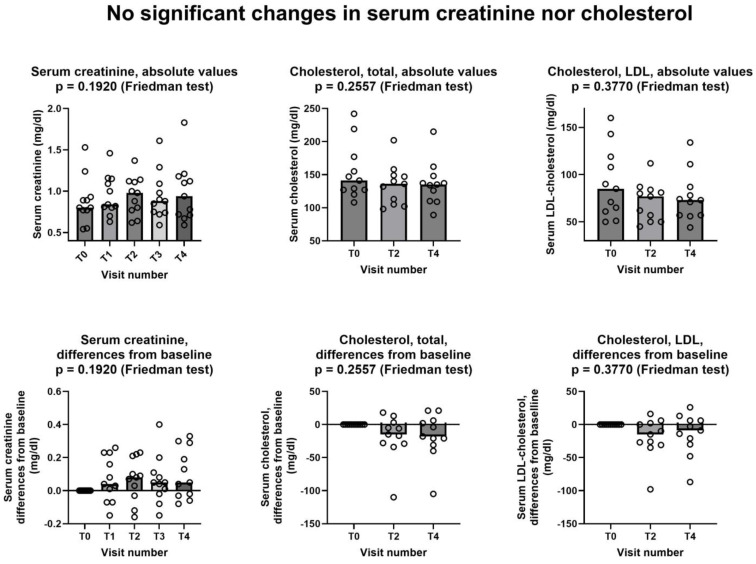
Lack of changes in serum creatinine, cholesterol, and LDL-cholesterol during the study. In all graphs, both individual values (empty circles) and median value at each visit (bars) are represented. Above row shows absolute values, below row shows differences from baseline. T0: baseline visit, before study procedures; T1 through T4: monthly visits, months 1 through 4. See text for additional details.

Figure 2 shows that no significant changes were observed in neither serum creatinine nor in serum cholesterol, either as total cholesterol or LDL-cholesterol.

### 3.3. Effects on Statin Myopathy

#### 3.3.1. Myopathy Score

**Figure 3:**
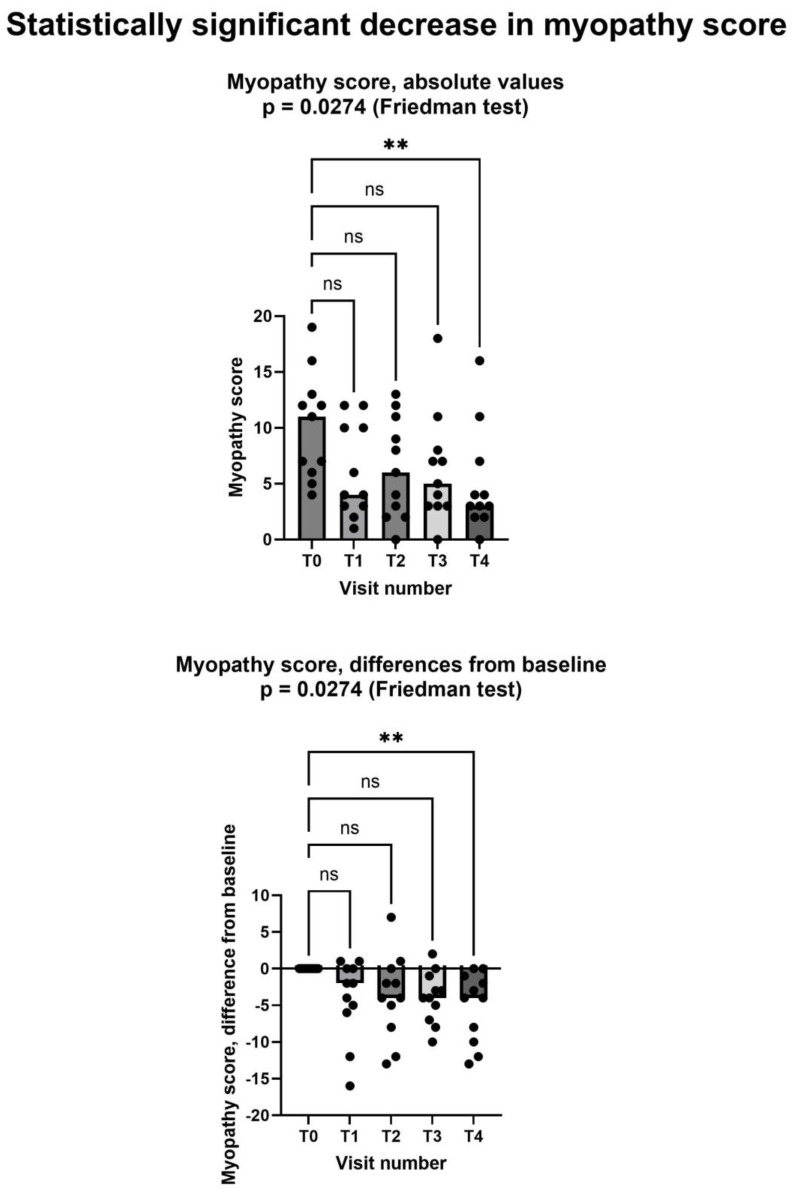
Shewmon and Craig’s “myopathy score” [10] significantly decreased after creatine supplementation, despite continued statin assumption. In both graphs, both individual values (filled circles) and median values at each visit (bars) are represented. Above graph shows absolute values, below one shows differences from baseline. Asterisks (**) show statistically significant difference in pairwise comparison (*p* = 0.0077, Dunn’s multiple comparisons test); ns = not significant. T0: baseline visit, before study procedures; T1 through T4: monthly visits, months 1 through 4. See text for additional details.

Figure 3 shows that Shewmon and Craig’s “myopathy score” [10] significantly decreased during the study period, with scores at the fourth month being significantly lower than at the baseline. The average decrease was −5.2 points between T0 and T4. It is noteworthy that in 6 out of the 11 patients, the decrease at T4 was greater than 50% compared to the baseline, representing a substantial and clinically significant decrease. No patient had a myopathy score higher at T4 than it was at T0.

**Figure 4:**
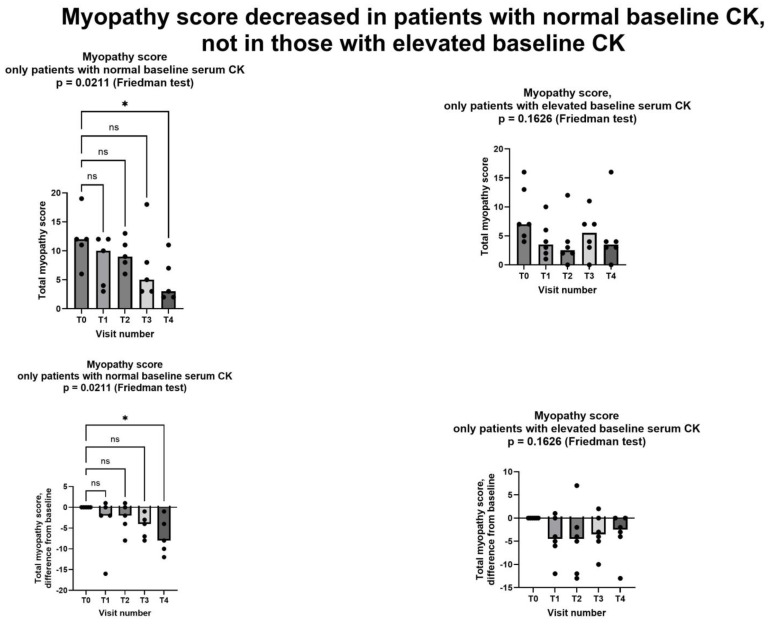
Myopathy score in patients with normal CK at baseline (left column) and in patients with elevated CK at baseline (right column). Only in patients with normal CK at baseline did we observe a statistically significant decrease in myopathy score. In all graphs, both individual values (filled circles) and median value at each visit (bars) are represented. Above graphs show absolute values, below ones show differences from baseline. Asterisk shows statistically significant difference in pairwise comparison (*p* = 0.0149, Dunn’s multiple comparisons test); ns = not significant. T0: baseline visit, before study procedures; T1 through T4: monthly visits, months 1 through 4. See text for additional details.

Since elevated baseline CK is probably a marker of more severe myopathy, we considered the effects of creatine supplementation separately in the five patients with and in the six patients without elevated serum CK at baseline. Shewmon and Craig’s myopathy score [10] decreased in a statistically significant way in patients with normal baseline CK, while the decrease did not reach statistical significant in those with abnormal baseline CK (Figure 4).

#### 3.3.2. Serum Creatine Kinase

**Figure 5:**
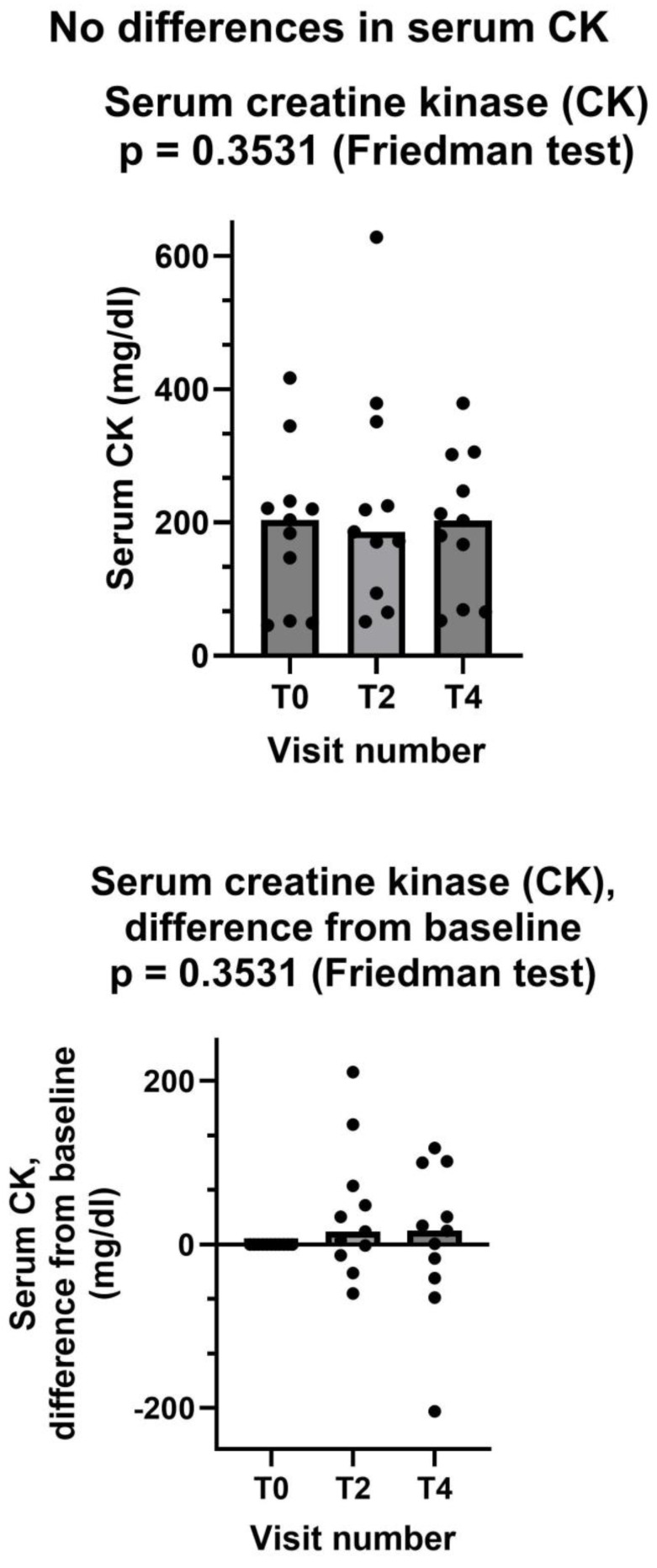
Creatine kinase did not decrease after creatine supplementation. In both graphs, both individual values (filled circles) and median values at each visit (bars) are represented. Above graph shows absolute values, below one shows differences from baseline. T0: baseline visit, before study procedures; T2 and T4: visits at months 2 and 4. See text for additional details.

By contrast, serum creatine kinase did not show any change after creatine supplementation (Figure 5). We should consider that only six patients has an abnormal CK value at the baseline. In these six patients, serum CK at the baseline was (mean ± standard deviation) 273 ± 87 mg/dL. At the end of the study, CK value was normalized in two patients but was still in the abnormal range in four patients. In one patient, serum CK was borderline but still within the normal range at the baseline (184 mg/dL), and it was abnormal (302 mg/dL) at the end of the study.

## 4. Discussion

This is a pilot study that was carried out to investigate the feasibility and possible effectiveness of low-dose creatine supplementation in statin myopathy. As a first endpoint, we tested whether at least half of the patients treated with creatine and the statin, to which they were intolerant, completed the study. The answer was positive; in fact, 11 out of 13 patients who entered the active phase of the study (85%) completed the study (Figure 1). We did not count in this computation the single patient that was withdrawn after enrollment but before starting study treatment; however, the percentage of study completion remains very high (79%) even if we include him. Of the few withdrawn patients, only one retired because of a creatine-related effect, i.e., increase in creatinine more than 1.5 times of its upper limit of normality (ULN). This effect is related to creatine because creatinine is the metabolite of creatine, thus it is prone to increase in the blood when blood creatine increases, as is the case during creatine supplementation [23]. It is important to emphasize that this increase is not necessarily an index of kidney damage; it might be simply the consequence of increased blood creatine, which in turn generates increased levels of creatinine. To precisely investigate renal function and its possible changes during creatine supplementation, methods not relying on creatinine dosage should be used, the main one being plasma clearance of 51Cr-EDTA [24,25]. Nevertheless, it is prudent (1) not to prescribe creatine supplementation to patients with impaired renal function and (2) to stop creatine supplementation should serum creatinine raise to 1.5–2 times its ULN [11]. However, it is important to note that in our study, which involved middle- and old-age patients, a very high percentage was able to complete the study without showing any significant creatinine increase. Additionally, the creatinine data of our patients (Figure 2) are in agreement with the literature data, including the literature review by two of us, showing that creatine supplementation does not cause kidney damage [11,26,27]. Finally, the fact that both total cholesterol and LDL-cholesterol did not increase during the study (Figure 2) confirms that, as one might have expected, the one-week stop of statin assumption at the beginning of the study does not adversely affect the level of cholesterol in the blood.

As for the two secondary endpoints, we met the first one, insofar as after creatinine supplementation. Shewmon and Craig’s “myopathy score” was significantly lower at T4 than at T0 (Figure 3). This is an important finding, because it strongly suggests that creatine supplementation may indeed mitigate statin myopathy, as previous data have already suggested [10,12,13]. By contrast, we did not meet the other secondary endpoint, because CK was not statistically different at T4 compared to T0 (Figure 5). It is important to note that CK elevation indicates structural damage to muscle cells, namely a membrane breakdown that allows intracellular CK to leak into the bloodstream [28]. Since not all patients with statin myopathy show elevated serum CK, and in fact statin myopathy can occur in the absence of clinically elevated CK [29], it is easy to hypothesize that muscle pain is probably an early symptom of statin-associated myopathy and that it initially occurs in the absence of structural damage to muscle cells, i.e., in the absence of CK serum elevation. In this early phase, our data strongly suggest that creatine supplementation may be capable of mitigating the clinical picture and perhaps of preventing further damage. By contrast, once structural muscle damage has occurred, i.e., after serum CK elevation, creatine supplementation is less effective (Figure 4).

It is important to note that the low-dose creatine we used was effective in reducing statin myopathy in patients without increases in serum CK. In fact, elderly patients sometimes have incomplete intestinal absorption of nutrients; thus, it is important to verify the effectiveness we saw.

As for the possible mechanism of action of creatine supplementation, we reviewed in our reference [13] data suggesting that statins may decrease in the muscle during the synthesis of creatine. Given the paramount importance of creatine for muscle metabolism and function, this may well be the rationale for their effectiveness in statin myopathy.

In the present study, we did not explore creatine supplementation beyond 4 months. It is possible that to maintain its effectiveness, creatine shall be supplemented for an unlimited time. However, creatine supplementation is safe even in the long term, as several studies have demonstrated (see, for example, those reviewed in our reference [11]).

The main limitations of our study are the limited number of subjects and the lack of untreated controls. Even with these limitations, our data strongly suggest that creatine supplementation may improve statin myopathy, especially in its milder and/or initial phase when serum CK is not elevated. Further research should try to confirm our data in a randomized, controlled trial.

## 5. Conclusions

Our data confirm previous reports showing that creatine supplementation is safe even in older subjects. Specifically, 2 g creatine t.i.d. for 1 week followed by 1 g. t.i.d. was well tolerated by most of our patients. Nevertheless, it is prudent not to administer it to patients with renal insufficiency and to monitor serum creatinine during supplementation.

Moreover, this low-dose creatine supplementation may be capable of significantly improving the symptoms of statin myopathy, especially in the milder and/or earlier stage when serum CK is not elevated.

Creatine supplementation may be a feasible option to allow the continuation of statin treatment, thus preventing or delaying the prescription of second-line anti-cholesterol treatments, whose high cost is problematic for developed countries and even more for emerging economies (see above, Table 1) [7].

Further research should be carried out to hopefully further confirm these findings.

## Figures and Tables

**Table 1:** Cost of some cholesterol-lowering treatments in Italy.

Drug	Monthly Cost [Reference]
Atorvastatin 80 mg/day	€13 [18]
Ezetimibe 10 mg/day	€59 [19] ^1^
Bempedoic acid 180 mg/day	€133 [20]
Alirocumab 75 mg	€716.26 [21]
Inclisiran 284 mg	€ (range) 776.64–1553.27 [22] ^2^

^1^ Based on the cost of €708/year. ^2^ Based on the cost of €4659.82 every 3 or every 6 months.

**Table 2:** Inclusion and exclusion criteria and preset criteria for study withdrawal.

Inclusion Criteria	Exclusion Criteria	Preset Criteria for Study Withdrawal
⁻ Diagnosis of statin-associated muscle symptoms, i.e., muscle symptoms occurring after exposure to one or more statins, having excluded other causes of muscle pain ⁻ Mild degree of such symptoms, e.g., muscle pain and/or weakness and/or cramps and/or CPK elevation < 5 upper limit of normal (ULN) ⁻ Age > 18 years ⁻ Indication for statin treatment for the prevention of cardiovascular disease and no contraindications to statin use	⁻ Current or past major muscle disease (e.g., rhabdomyolysis or severe myositis), statin-related or not. ⁻ Hypothyroidism ⁻ Autoimmune diseases ⁻ Kidney insufficiency, as evidenced by elevated serum creatinine	⁻ Withdrawal of patient consent at any time ⁻ Any medical condition that may affect the patient’s safety in continuing the study ⁻ Non-adherence to creatine and/or statin therapy ⁻ Changes in statin treatment during the study ⁻ Increase in creatinine value by more than 1.5 its upper limit of normality (ULN).

## Data Availability

The whole database is provided as Supplementary Materials to this article.

## Supplementary Materials

The following supporting information can be downloaded at: https://www.mdpi.com/article/10.3390/jcm13237194/s1, Table S1: Descriptive statistics; Table S2: Full database, raw data.
