# Supplementary material for: Low-Dose Creatine Supplementation May Be Effective in Early-Stage Statin Myopathy: A Preliminary Study
Source: J Clin Med. 2024 Nov 27;13(23):7194. doi: 10.3390/jcm13237194 (PMC11642150; doi:10.3390/jcm13237194)
Supplement: Supplementary file 1 [file jcm-13-07194-s001.zip › S2.pdf]

CK			
	T0	T2	T4
pt 1	221	186	180
pt 2	N/A	51	53
pt 3	49	65	66
pt 4	345	379	379
pt 5	184	171	302
pt 6	232	172	167
pt 7	220	225	203
pt 8	46	94	69
pt 9	204	351	306
pt 10	147	219	247
pt 11	417	628	213

Total cholesterol			
	T0	T2	T4
pt 1	242	132	137
pt 2	129	147	135
pt 3	127	98	109
pt 4	141	136	162
pt 5	120	105	89
pt 6	127	140	148
pt 7	108	102	110
pt 8	219	202	215
pt 9	147	113	126
pt 10	177	149	137
pt 11	155	158	134

LDL-cholesterol			
	T0	T2	T4
pt 1	160	62	73
pt 2	51	57	57
pt 3	71	45	57
pt 4	85	87	111
pt 5	65	50	44
pt 6	61	77	74
pt 7	50	50	56
pt 8	143	112	134
pt 9	108	81	78
pt 10	119	84	71
pt 11	90	80	87

Serum creatinine					
	T0	T1	T2	T3	T4
pt 1	0.55	0.63	0.62	0.59	0.59
pt 2	0.54	0.70	0.64	0.74	0.67
pt 3	0.89	1.15	1.11	1.29	1.18
pt 4	0.93	1.16	1.14	0.98	1.12
pt 5	1.53	1.46	1.37	1.61	1.83

pt 6	0.80	0.84	0.88	0.85	0.78
pt 7	1.24	1.09	1.12	1.09	1.21
pt 8	0.77	0.8	0.8	0.75	0.71
pt 9	0.89	0.82	0.98	0.90	0.94
pt 10	0.8	0.81	0.77	0.72	0.72
pt 11	0.77	1	1	0.88	1.10

Shewmon and Craig's "myopathy score"					
	T0	T1	T2	T3	T4
pt 1	16	10	4	11	16
pt 2	12	12	13	8	11
pt 3	6	4	6	3	2
pt 4	4	4	2	0	4
pt 5	19	3	11	18	7
pt 6	7	3	2	7	4
pt 7	7	2	3	4	3
pt 8	12	10	8	5	2
pt 9	13	1	0	3	0
pt 10	11	12	9	3	3
pt 11	5	6	12	7	3
