# Supplementary material for: Low-Dose Creatine Supplementation May Be Effective in Early-Stage Statin Myopathy: A Preliminary Study
Source: J Clin Med. 2024 Nov 27;13(23):7194. doi: 10.3390/jcm13237194 (PMC11642150; doi:10.3390/jcm13237194)
Supplement: Supplementary file 1 [file jcm-13-07194-s001.zip › S1.pdf]

Serum creatinine (mg/dl)

	T0	T1	T2	T3	T4
25% Percentile	0.77	0.8	0.77	0.74	0.71
Median	0.8	0.84	0.98	0.88	0.94
75% Percentile	0.93	1.2	1.1	1.1	1.2

Serum creatinine. difference from baseline (mg/ml)

	T0	T1	T2	T3	T4
25% Percentile	0	-0.07	-0.03	-0.02	-0.03
Median	0	0.04	0.08	0.05	0.05
75% Percentile	0	0.23	0.21	0.11	0.29

Total serum cholesterol (mg/dl)

	T0	T2	T4
25% Percentile	127	105	110
Median	141	136	135
75% Percentile	177	149	148

Serum cholesterol. difference from baseline (mg/ml)

	T0	T2	T4
25% Percentile	0	-29	-31
Median	0	-15	-18
75% Percentile	0	3	6

Serum LDL-cholesterol (mg/dl)

	T0	T2	T4
25% Percentile	61	50	57
Median	85	77	73
75% Percentile	119	84	87

Serum LDL-cholesterol. difference from baseline (mg/ml)

	T0	T2	T4
25% Percentile	0	-31	-30
Median	0	-15	-9
75% Percentile	0	2	6

Myopathy score

	T0	T1	T2	T3	T4
25% Percentile	6	3	2	3	2
Median	11	4	6	5	3
75% Percentile	13	10	11	8	7

Myopathy score, difference from baseline

	T0	T1	T2	T3	T4
25% Percentile	0	-6	-8	-7	-10
Median	0	-2	-4	-4	-4
75% Percentile	0	0	0	-1	-1

Myopathy score, only patients with normal baseline CK

	T0	T1	T2	T3	T4
25% Percentile	8.5	3.5	7	3	2
Median	12	10	9	5	3
75% Percentile	16	12	12	13	9

Myopathy score, differences from baseline only patients with normal baseline CK

	T0	T1	T2	T3	T4
25% Percentile	0	-9	-6	-7.5	-11
Median	0	-2	-2	-4	-8
75% Percentile	0	0.5	0.5	-2	-2.5

Myopathy score, only patients with elevated baseline CK

	T0	T1	T2	T3	T4
25% Percentile	4.8	1.8	1.5	2.3	2.3
Median	7	3.5	2.5	5.5	3.5
75% Percentile	14	7	6	8	7

Myopathy score, differences from baseline only patients with elevated baseline CK

	T0	T1	T2	T3	T4
25% Percentile	0	-7,5	-12	-6,3	-6,3
Median	0	-4,5	-4,5	-3,5	-2,5
75% Percentile	0	0,25	0,25	0,5	0

Serum creatine kinase (mg/dl)

	T0	T2	T4
25% Percentile	52	94	69
Median	204	186	203
75% Percentile	232	351	302

Serum creatine kinase, difference from baseline (mg/dl)

	T0	T2	T4
25% Percentile	0	-13	-41
Median	0	16	17
75% Percentile	0	72	100
